# Spindle-like MIL101(Fe) decorated with Bi_2_O_3_ nanoparticles for enhanced degradation of chlortetracycline under visible-light irradiation

**DOI:** 10.3762/bjnano.13.91

**Published:** 2022-09-28

**Authors:** Chen-chen Hao, Fang-yan Chen, Kun Bian, Yu-bin Tang, Wei-long Shi

**Affiliations:** 1 School of Environmental and Chemical Engineering, Jiangsu University of Science and Technology, Zhenjiang, 212100, PR China; 2 School of Materials Science and Engineering, Jiangsu University of Science and Technology, Zhenjiang, 212100, PR China

**Keywords:** Bi_2_O_3_, chlortetracycline, metal–organic frameworks, MIL101(Fe), photocatalysts, Z-scheme heterojunction

## Abstract

Improving the photocatalytic performance of metal–organic frameworks (MOFs) is an important way to expand its potential applications. In this work, zero-dimensional (0D) Bi_2_O_3_ nanoparticles were anchored to the surface of tridimensional (3D) MIL101(Fe) by a facile solvothermal method to obtain a novel 0D/3D heterojunction Bi_2_O_3_/MIL101(Fe) (BOM). The morphology and optical properties of the as-prepared Bi_2_O_3_/MIL101(Fe) composite were characterized. The photocatalytic activity of the synthesized samples was evaluated by degrading chlortetracycline (CTC) under visible-light irradiation. The obtained BOM-20 composite (20 wt % Bi_2_O_3_/MIL101(Fe)) exhibits the highest photocatalytic activity with CTC degradation efficiency of 88.2% within 120 min. The degradation rate constant of BOM-20 toward CTC is 0.01348 min^−1^, which is 5.9 and 4.3 times higher than that of pristine Bi_2_O_3_ and MIL101(Fe), respectively. The enhanced photocatalytic activity is attributed to the formation of a Z-scheme heterojunction between Bi_2_O_3_ and MIL101(Fe), which is conducive to the rapid separation of photogenerated carriers and the enhancement of photogenerated electron and hole redox capacity. The intermediate products were analyzed by liquid chromatography–mass spectrometry (LC–MS), and a possible photocatalytic degradation path of CTC was proposed. This work provides a new perspective for the preparation of efficient MOF-based photocatalysts.

## Introduction

Tetracyclines, as the second most widely used antibiotic in the world, have been widely applied in clinics, aquaculture, and livestock due to its broad-spectrum antibacterial properties and low price [1–2]. Chlortetracycline (CTC) is the first tetracycline antibiotic used for veterinary purposes [3]. Due to the abusive use of CTC in livestock industry and the low absorption in animals, a large amount of CTC has been released into the environment through animal excretions. At present, CTC has been detected in aquatic environments in concentrations of approx. 0.08–0.61 μg/L [3]. The residual CTC in the environment can lead to the emergence of antibiotic-resistant pathogens, posing a serious threat to human health and aquatic ecosystems [4]. So, it is an important and urgent task to find an efficient and green method to remove CTC existing in the aquatic environment. Up to now, various technologies, including adsorption, hydrolysis, and biodegradation, have been applied in the removal of pollutants from water [5–8]. Owing to the relatively complicated treatment, high cost, and possible secondary pollution, these traditional measures were difficult to be popularized. Photocatalysis is considered as an alternative and promising strategy for degradation and mineralization of antibiotics due to its low cost, high efficiency, mild reaction condition, and no secondary pollution [9–11]. A variety of semiconductors have been used for antibiotic degradation [12–17]. However, poor adsorptive ability, low quantum yield, and rapid recombination of photogenerated carriers restrict the real applications of photocatalysis in the removal of CTC from wastewater [18]. Thus, it is still crucial to develop efficient photocatalysts for CTC degradation.

Metal–organic frameworks (MOFs) are a kind of micro- or mesoporous materials established by the self-assembly of organic linkers and metal-cluster or metal-ion nodes [19]. The MOF materials possess large surface areas, high pore volume, tunability, uniform cavities, and excellent thermal stability [20–21]. These advantages make it appalling to adsorption [22], gaseous capture/separation [23], sensing [24], and drug release applications [25]. Moreover, some MOFs can be excited under UV or visible light and exhibit light harvesting properties due to ligand–metal charge transfer (LMCT). For this reason, these MOFs are considered as emerging semiconductor-like photocatalysts and attention is growing toward these materials [26–29]. In 2007, Garcia and coworkers have first reported photocatalytic degradation of phenol by using MOF-5 as a photocatalyst under UV light irradiation [30]. So far, a large number of MOFs have been shown to exhibit photocatalytic activity in H_2_ production, organic pollutant degradation, and Cr(VI) and CO_2_ reduction [26–27,31–33].

Among MOF catalysts, MIL101(Fe) is a cage-like structure formed by self-assembly of iron and bridged terephthalic acid molecules. It has high hydrothermal stability, low cost, good hydrophilicity, non-toxicity, and environmental friendliness [20,34]. Most importantly, MIL101(Fe) contains abundant iron-oxo (Fe-O) clusters, which makes it a photocatalyst with visible-light response [19,21,35]. However, pure phase MIL101(Fe), like most semiconductor photocatalysts, has inherent defects, such as low conductivity and high recombination efficiency of photogenerated electron–hole pairs [26,36]. To overcome these shortcomings, several strategies have been developed. One approach is to use carbon nanotubes or carbon quantum dots to modify MIL101(Fe) to enhance its conductivity and broaden its visible-light response [37–38]. Another strategy is to construct MIL101-based heterostructures with the aid of narrow-gap semiconductors to promote the separation and transfer of photogenerated charges and resultant photocatalytic activity [26]. Up to date, a few semiconductors such as ZnO [39], TiO_2_ [19] and g-C_3_N_4_ [40] have been employed to construct heterojunctions with MIL101(Fe). The abovementioned MIL101-based heterojunctions are all traditional heterojunctions which promote the separation of electron–hole pairs, thus improving the photocatalytic activity of MIL101(Fe) to a certain extent. Nevertheless, the redox ability of the catalyst is weakened due to the fact that the reduction and oxidation processes on the catalyst surface occur at lower oxidation and reduction potentials, respectively [41]. In recent years, artificial Z-scheme heterojunction catalysts have generated extensive interest since its special electronic structure not only promotes separation of electron–hole pairs but also remains with high redox capacity [42]. Therefore, the photocatalytic activity of MIL101(Fe) can be improved by combining it with other suitable semiconductor materials to construct Z-scheme heterojunctions.

Bismuth trioxide (Bi_2_O_3_), a metal oxide semiconductor with a bandgap of 2.8 eV, can be excited by visible light [43–44]. However, pure Bi_2_O_3_ exhibits poor photocatalytic activity due to the narrow response region to visible light and fast recombination of photoproduced charge carriers [45]. To improve the photocatalytic activity of Bi_2_O_3_, many researchers have attempted to modify Bi_2_O_3_. Since Bi_2_O_3_ has a relatively positive valence band position, combing Bi_2_O_3_ with other semiconductors to construct Bi_2_O_3_-based heterojunctions has attracted extensive interest. A large number of Bi_2_O_3_-based heterojunctions, such as Bi_2_O_3_/g-C_3_N_4_ [46], WO_3_/g-C_3_N_4_/Bi_2_O_3_ [47], Bi_2_O_3_/BiOCl [48], Bi_2_O_3_/ZnO [49], and Bi_2_O_3_/BiVO_4_ [50] have been synthesized. However, to the best of our knowledge, there is no report about the construction of Z-scheme heterojunctions by coupling MIL101(Fe) and Bi_2_O_3_.

Herein, in order to enhance the photocatalytic efficiency of MIL101(Fe) for degradation of CTC, a novel 0D/3D heterojunction catalyst Bi_2_O_3_/MIL101(Fe) was prepared by anchoring Bi_2_O_3_ nanoparticles to the surface of MIL101(Fe). The experimental results show that the recombination of photogenerated carriers in Bi_2_O_3_/MIL101(Fe) is effectively inhibited, and the photocatalytic activity of the composite is significantly improved compared with that of a monomer catalyst. At the same time, the results of the capture experiment and electron spin resonance (ESR) experiment suggest that the electron transfer path between Bi_2_O_3_ and MIL101(Fe) accords with the Z-type transfer mechanism. The possible photocatalytic degradation pathways were investigated via the analysis of the intermediate products in the degradation process of CTC.

## Materials and Methods

### Preparation of photocatalysts

**The preparation of Bi_2_O_3_.** Briefly, 5 g of Bi (NO_3_)_3_·5H_2_O was added into a crucible, then heated to 520 °C at a heating rate of 5 °C/min, and maintained for 2 h. Next, it was naturally cooled to room temperature and the resulting yellow bulks were ground to obtain Bi_2_O_3_ nanoparticles.

**Scheme 1:**
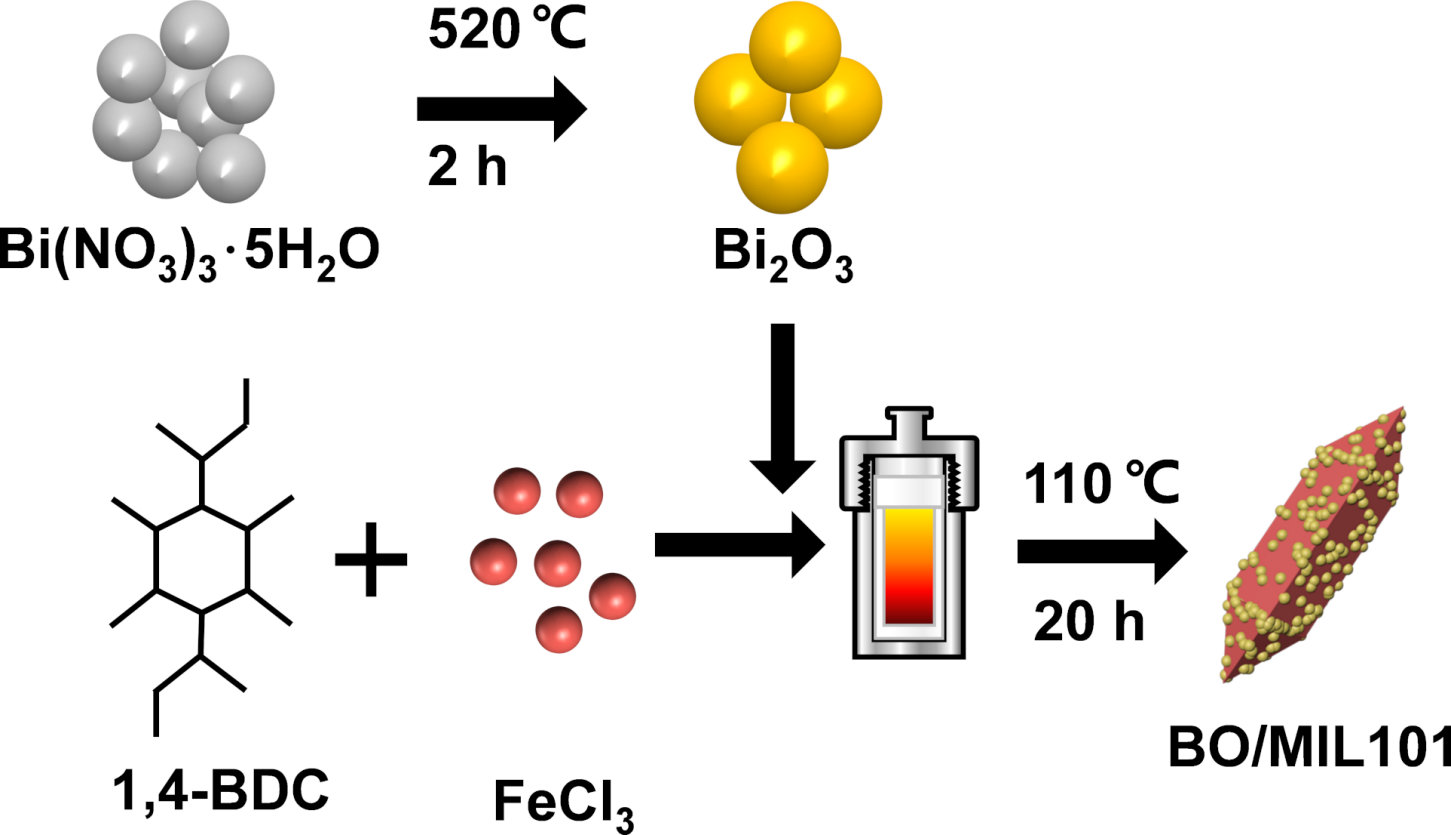
Schematic illustration of the synthetic process of Bi_2_O_3_/MIL101(Fe) heterojunction.

**The preparation of Bi_2_O_3_/MIL101(Fe).** The Bi_2_O_3_/MIL101(Fe) composite was fabricated by a solvothermal method. The schematic synthesis procedure of Bi_2_O_3_/MIL101(Fe) is illustrated in Scheme 1. In a manner analogous to a previous report [35], 1.35 g of FeCl_3_·6H_2_O and 0.412 g of 1,4-benzenedicarboxulic acid (H_2_BDC) were added to 30 mL of dimethylformamide (DMF) under sonication to form a transparent brown solution. Subsequently, as-synthesized Bi_2_O_3_ nanoparticles were dispersed into the above solution by sonication for 40 min. The mixture was placed in a Teﬂon-lined steel autoclave and heated at 110 °C for 24 h. The orange solid product collected by centrifugation was rinsed by deionized water and ethanol for several times to remove unreacted substances, dried at 60 °C for 10 h in a vacuum oven, and ground to obtain Bi_2_O_3_/MIL101(Fe) composites. The as-prepared Bi_2_O_3_/MIL101(Fe) in different mass ratios of Bi_2_O_3_ of 5, 10, 20, and 30 wt % were labeled as BOM-5, BOM-10, BOM-20 and BOM-30, respectively. For comparison, MIL101(Fe) was prepared with the same experimental conditions and route as the Bi_2_O_3_/MIL101(Fe) composite without the addition of Bi_2_O_3_ nanoparticles.

### Photocatalytic test

The photocatalytic activity of the prepared samples was evaluated by the degradation of chlortetracycline (CTC) under visible-light irradiation. The test method is similar to what has been described in [35]. In a typical reaction, 30 mg of photocatalysts were added to 100 mL of a CTC solution with an initial concentration of 20 mg/L and stirred in the dark for 30 min to reach adsorption–desorption equilibrium. Then, the photocatalytic reaction was initiated by a 300 W xenon lamp with a 420 nm cut-off filter. At 20 min intervals, 4 mL of the sample was taken out and centrifuged to remove photocatalysts. The concentration of CTC in the supernatant was determined by UV–vis spectrophotometry at a wavelength of 357 nm. The degradation rate can be calculated by Equation 1:


[1] $$\text{Degradation }\left(\%\right)=\left(1-C_{t}/C_{0}\right)\times 100\%,$$

where *C*_0_ and *C_t_* represent the concentration of CTC at the initial time and time *t*, respectively.

### Characterization of the as-prepared catalyst

The crystalline structure of the prepared photocatalyst was analyzed by X-ray diffraction spectrometry (Empyrean, Panalytical, Holland) with Cu Kα radiation at a scanning speed of 7 °/min. The morphology of the samples was observed by scanning electron microscopy (SEM, FEI-quanta 200, Japan Electronics, Japan), transmission electron microscopy (TEM, FEI-Tecnai F20, USA) and high-resolution transmission electron microscopy (HRTEM, JEOL 2100F, Japan). The element valence and chemical composition was investigated using X-ray photoelectron spectroscopy (XPS, Axis ultra-DLD, England). The Brunauer–Emmett–Teller (BET) surface area and pore size were determined using N_2_ adsorption–desorption isotherms (Microfor TriStar II Plus 2.02 system, USA). The UV–vis diffuse reflectance spectra (UV–vis DRS) were recorded by UV–vis spectrophotometry (FTS-165, PerkinElmer, USA) with BaSO_4_ as a reference. The photoluminescence spectrum (PL) was obtained from a luminescence spectrometer at an excitation wavelength of 446 nm (RF-530IPC, Shimadzu, Japan). The photocurrent response and electrochemical impedance spectra (EIS) were measured by an electrochemical workstation (CHIQ660D, Chenhua Instrument, China). Electronic paramagnetic resonance signals were recorded with an electron paramagnetic resonance spectrometer (Brooke A300, Germany).

## Results and Discussion

### Characterization

**Figure 1:**
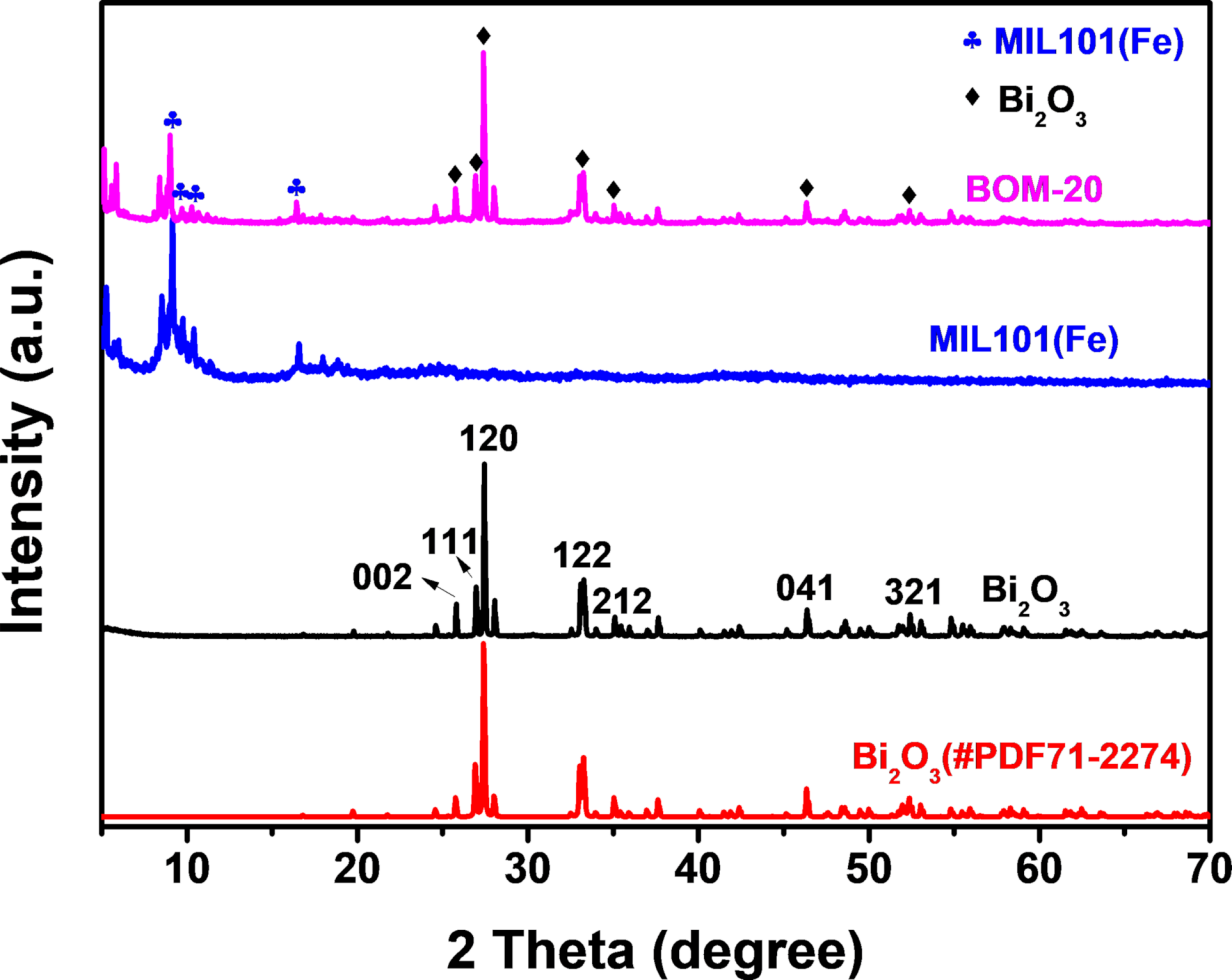
XRD patterns of MIL101, Bi_2_O_3_, and BOM-20.

Figure 1 shows the XRD pattern of the synthesized MIL101(Fe), Bi_2_O_3_, and BOM-20 composite.

The characteristic diffraction peaks of MIL101(Fe) at 8.9°, 10.2°, 10.6° and 16.4° were observed, which is in good accordance with previous reports [37,51], indicating that MIL101(Fe) was successfully prepared. In the XRD pattern of the prepared Bi_2_O_3_ sample, peaks at 2θ = 25.88°, 26.87°, 27.37°, 33.15°, 35.12°, 46.52°, and 52.56° correspond to the (002), (111), (120), (122), (212), (041), and (321) plane of monoclinic α-Bi_2_O_3_, which matches well with the standard card α-Bi_2_O_3_ (PDF#71-2274) [52], suggesting good crystallization of the prepared Bi_2_O_3_. The characteristic diffraction peaks of MIL101(Fe) and Bi_2_O_3_ are all found in the XRD pattern of BOM-20, indicating the existence of MIL101(Fe) and Bi_2_O_3_ in BOM-20. Additionally, the presence of characteristic peaks of MIL101(Fe) in the BOM-20 composite demonstrates that the framework of MIL101(Fe) is unchanged after loading the Bi_2_O_3_ nanoparticles.

**Figure 2:**
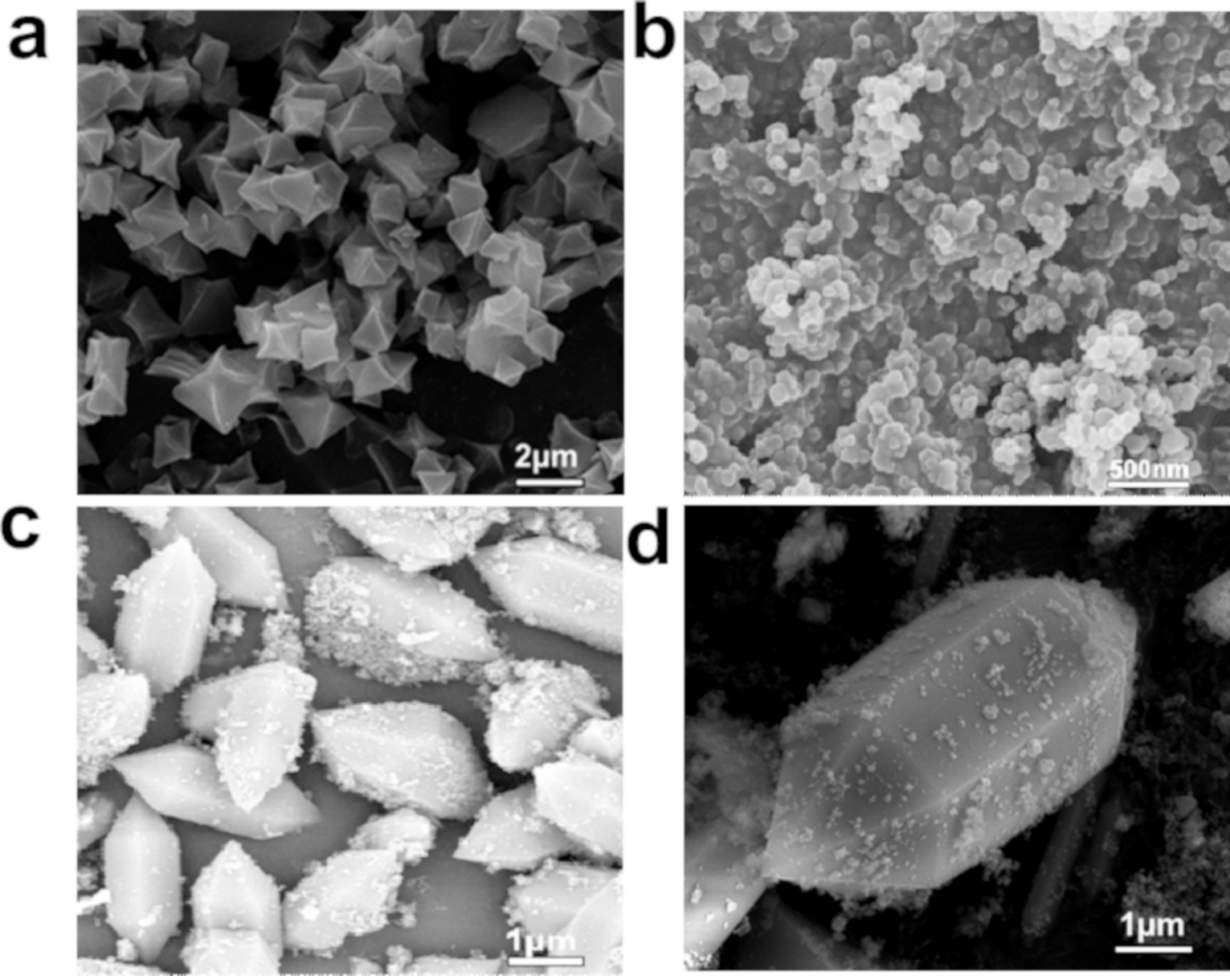
SEM images of (a) MIL101(Fe), (b) Bi_2_O_3_, and (c,d) BOM-20.

The morphology and microstructure of Bi_2_O_3_, MIL101(Fe), and BOM-20 were observed by SEM, TEM, and HRTEM. Figure 2 shows SEM images of Bi_2_O_3_, MIL101(Fe), and BOM-20. Figure 2a reveals that MIL101(Fe) appears as an octahedron with a smooth surface and size of approx. 1–2 μm, which is consistent with a previous report [53]. As shown in Figure 2b, pristine Bi_2_O_3_ shows nanoparticles with diameters of approx. 10–20 nm. In the SEM image of the composite BOM-20 (Figure 2c), Bi_2_O_3_ nanoparticles are tightly attached on the surface of MIL101(Fe). Note that MIL101(Fe) in BOM-20 presents a spindle-like shape instead of an octahedron shape, and its size increases compared with that of pristine MIL101(Fe). In previous reports, graphene quantum dots changed the balance of organic ligands, which led to the change of MIL101(Fe) size [38]. In addition, Liao et al. reported that morphology or size of MOFs are dependent on nucleation rate, (i.e., slow nucleation could increase crystal growth and as a result produce large-sized particles [54]). As a result, Bi_2_O_3_ nanoparticles affect the shape and size of MIL101(Fe) and this may be due to the addition of Bi_2_O_3_ to the precursor, altering the balance of ligands and simultaneously slowing the nucleation rate of MIL101(Fe).

**Figure 3:**
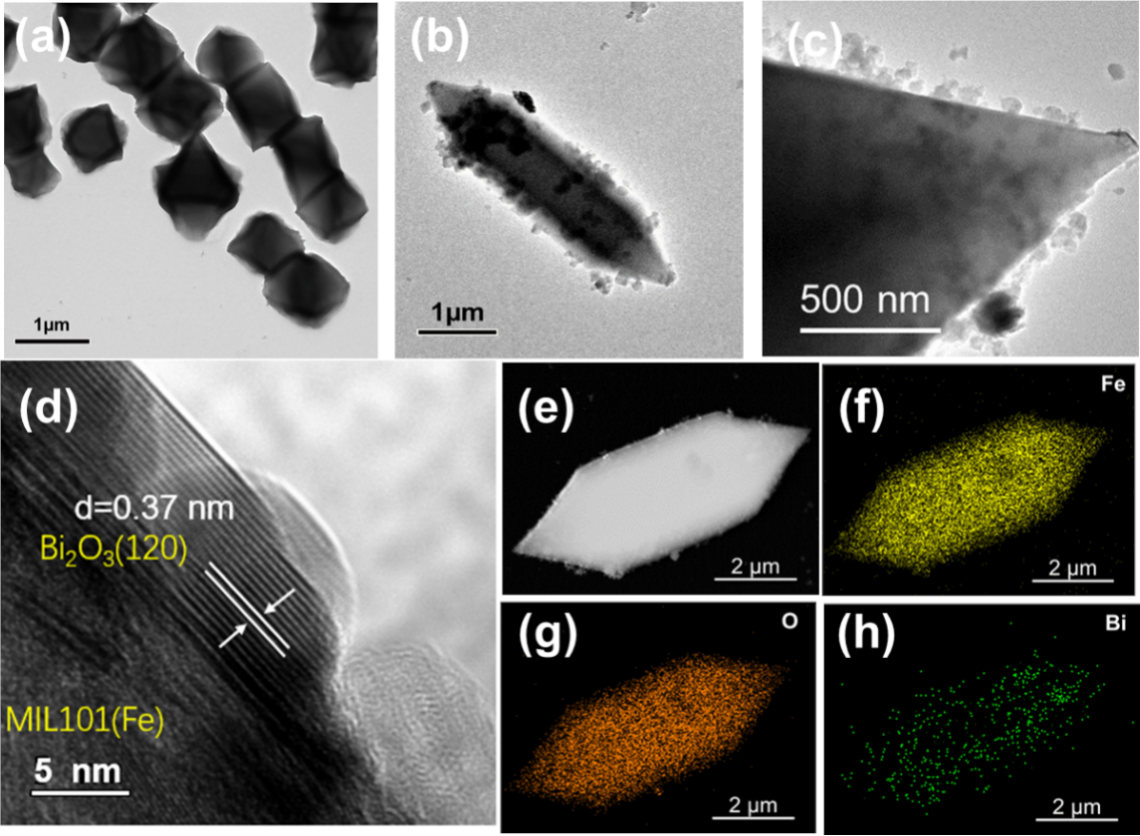
TEM image of (a) MIL101(Fe) and (b, c) BOM-20. (d) HRTEM image of BOM-20. (e) HAADF-STEM image of BOM-20 and the corresponding elemental maps of (f) Fe, (g) O, and (h) Bi.

To verify the formation of a heterojunction between MIL101(Fe) and Bi_2_O_3_, TEM and HRTEM images were obtained. As seen in Figure 3b and Figure 3c, TEM images of BOM-20 confirm that tiny Bi_2_O_3_ nanoparticles closely and uniformly adhere to the surface of MIL101(Fe). Figure 3d shows an HRTEM image of BOM-20 composites. The lattice fringes of 0.37 nm correspond to the (120) facet of α-Bi_2_O_3_ [55]. Meanwhile, the tight contact interface between Bi_2_O_3_ and MIL101(Fe) can be clearly observed. Furthermore, the elemental mapping images of BOM-20 (Figure 3e–h) reveal the homogeneous distribution of Bi in the entire BOM-20 composite, which further confirms that Bi_2_O_3_ nanoparticles are uniformly loaded on the surface of MIL101(Fe). Results of SEM and TEM provided convincing evidence for the successful construction of heterojunctions with a 0D/3D structure between Bi_2_O_3_ nanoparticles and tridimensional MIL101(Fe). This 0D/3D heterojunction is conducive to fast separation and migration of the photoproduced electron–hole pairs due to more contact areas between Bi_2_O_3_ and MIL101(Fe) and shorter transfer distances of photoinduced charge carriers [8], which results in enhanced photocatalytic activity of the Bi_2_O_3_/MIL101(Fe) composite.

**Figure 4:**
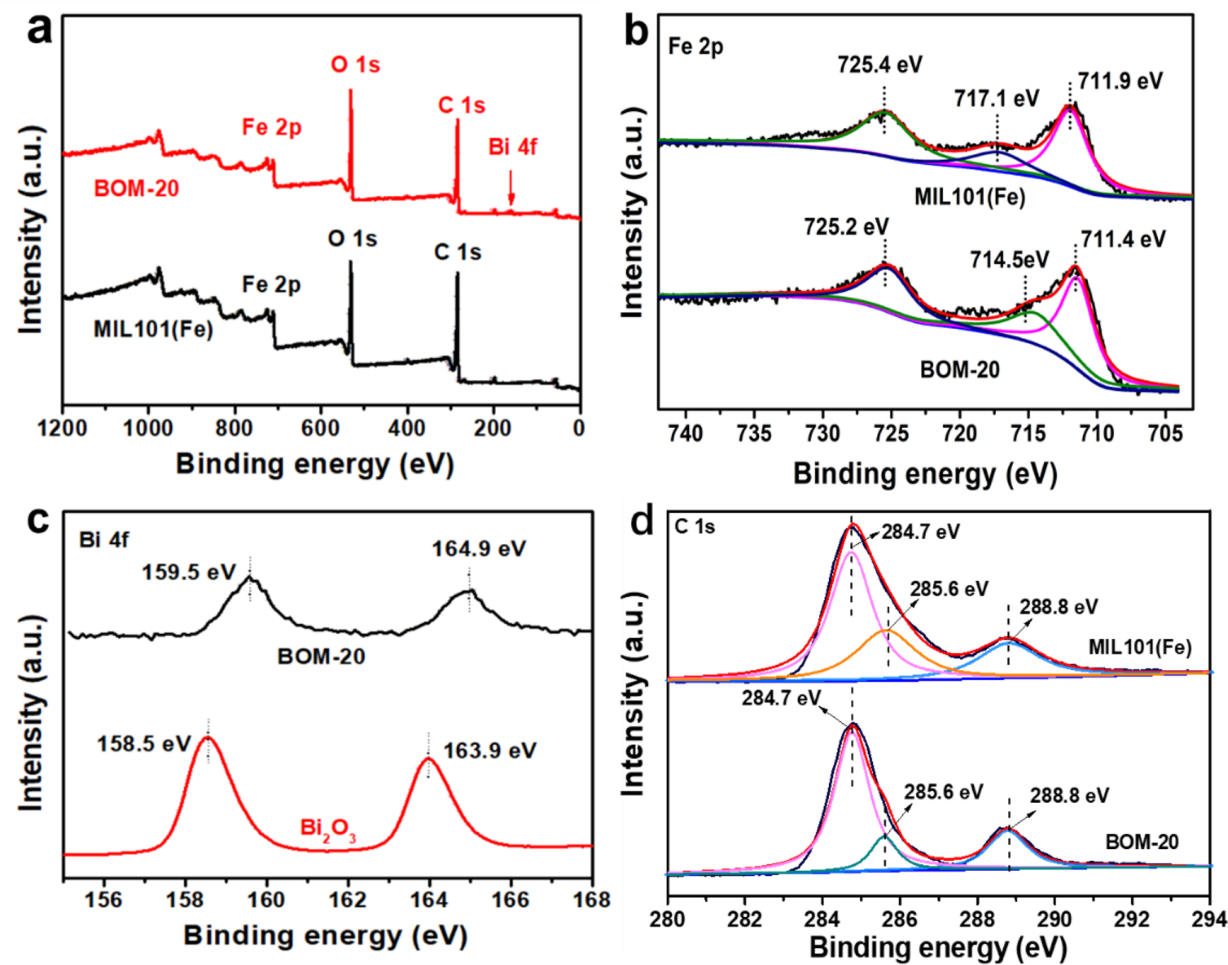
(a) XPS survey spectra of BOM-20 and high-resolution XPS spectra of (b) Fe 2p; (c) Bi 4f; and (d) C 1s.

X-ray photoelectron spectroscopy was performed to explore the elemental compositions and the surface chemical states of BOM-20 and the results are presented in Figure 4. The XPS survey spectrum (Figure 4a) indicates the existence of Fe, Bi, O, and C in BOM-20 composites. In the high-resolution spectrum of Fe 2p (Figure 4b), peaks at 725.2 and 711.4 eV are ascribed to Fe 2p_1/2_ and 2p_3/2_, respectively, implying the existence of Fe–O bonds [37]. The difference of binding energy between these two peaks is 13.9 eV, suggesting the presence of Fe^3+^ in BOM-20 [56]. Furthermore, the satellite peak of Fe(III) at 714.5 eV is observed, further indicating the presence of Fe^3+^ in BOM-20 [38]. However, the characteristic peak at 714.5 eV of Fe^3+^ in the BOM-20 composite was shifted toward lower binding energy relative to MIL101(Fe) (717.1eV), indicating an increased electron density on Fe^3+^ [42,57]. As shown in Figure 4c, the Bi 4f spectra of BOM-20 were fitted by two peaks at 159.5 and 164.9 eV, which are assigned to Bi 4f_7/2_ and Bi 4f_5/2_, respectively, corroborating the presence of Bi^3+^ [52,57]. The binding energies of Bi 4f at 159.5 and 164.9 eV of BOM-20 are higher than those of pristine MIL101(Fe), which indicates a decreased electron density on Bi. In BOM-20, the increased electron density on Fe^3+^ and the decreased electron density on Bi may be attributed to the interaction between Bi_2_O_3_ and MIL101(Fe), which further confirms the formation of Bi_2_O_3_/MIL101(Fe) heterojunction [57]. In the C 1s spectrum (Figure 4d), peaks at 284.8, 285.6, and 288.8 eV can be assigned to the carbon atom bond (C–C) in the benzoic rings and carbon organic linkers (C–O and O–C=O) of the H_2_BDC ligand, respectively [37].

### Optical and electronic properties

**Figure 5:**
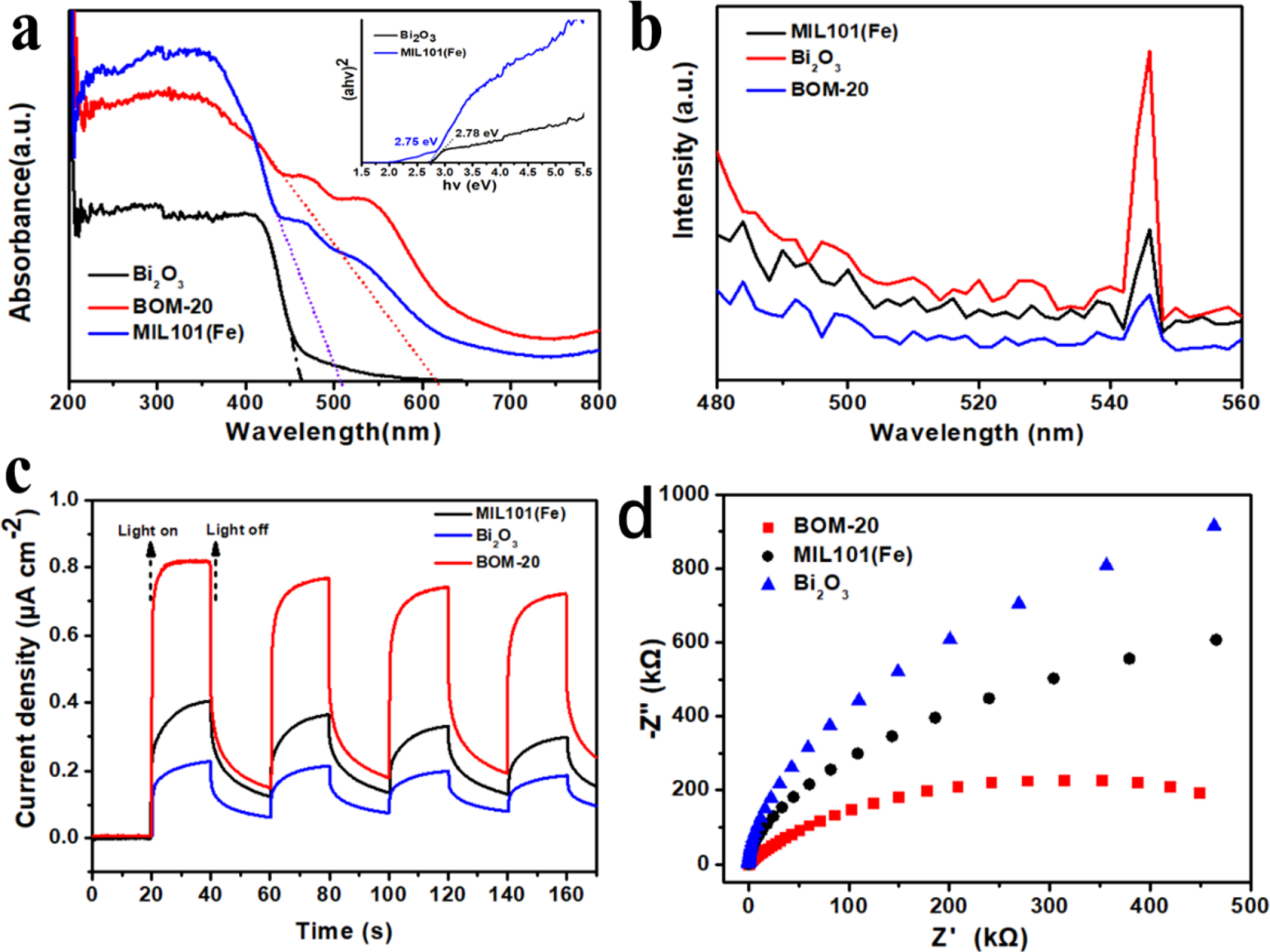
(a) UV–vis spectra, (b) PL spectra, (c) transient photocurrent responses, and (d) EIS spectra of MIL101(Fe), Bi_2_O_3_, and BOM-20.

To investigate the optical response and bandgap of the prepared samples, UV–vis diffuse reflectance (UV–vis DRS) spectra were recorded and given in Figure 5.

As seen from Figure 5a, both Bi_2_O_3_ and MIL101(Fe) show strong visible-light response with absorption edge of 460 nm and 510 nm, respectively. Compared with MIL101(Fe) and Bi_2_O_3_, the absorption edge of BOM-20 significantly red shifts to approx. 620 nm. MIL101(Fe) and Bi_2_O_3_ show a synergistic effect in improving visible-light absorption of the BOM-20 composite, which improves the photocatalytic activity due to the generation of more photoinduced electrons and holes. The bandgap (*E*_g_) of a semiconductor is usually estimated by the Tauc formula (α*h*ν) = *A*(*h*v − *E*_g_)*^n^*^/2^, where α is the absorbance, *h*ν is the photon energy, *A* is a constant, *E*_g_ is the bandgap , and *n* is a constant. For Bi_2_O_3_ and MIL101(Fe), as direct bandgap semiconductors, the value of *n* is 1 [58]. The plots of (α*h*ν)^2^ as a function of (*h*ν) for Bi_2_O_3_ and MIL101(Fe) are shown in the insert of Figure 5a. According to that, the bandgap of Bi_2_O_3_ and MIL101(Fe) are 2.78 and 2.75 eV, respectively.

In order to explore the separation of photoproduced electron–hole pairs in BOM-20, PL spectra were measured at an excitation wavelength of 464 nm. Generally, the weaker PL emission, the lower the recombination efficiency of electron–hole pairs [8]. As displayed in Figure 5b, Bi_2_O_3_ possesses a strong PL emission peak at approximately 550 nm, which is due to the rapid recombination of photogenerated electron–hole pairs in Bi_2_O_3_. MIL101(Fe) and BOM-20 composite show similar emission peaks at 546 nm. Moreover, BOM-20 peak intensity is obviously weaker than that of MIL101(Fe) and Bi_2_O_3_, which indicates that the recombination of photogenerated electron–hole pairs was effectively inhibited in BOM-20.

To further elucidate the transfer behavior of the charge carriers in BOM-20, photocurrent responses were carried out under visible light (λ > 420 nm) and were displayed in Figure 5c. The photocurrent density of the BOM-20 composite is significantly higher than that of pristine Bi_2_O_3_ and MIL101(Fe), which suggests that the formation of the heterojunction can promote charge transfer on the interface between MIL101(Fe) and Bi_2_O_3_. Furthermore, the interfacial charge transfer behavior of the BOM-20 composite is also confirmed by EIS. In Figure 5d, BOM-20 exhibits the smallest arc radius, meaning that BOM-20 possesses the lowest resistance and the highest separation rate of electron–hole pairs on the interface of MIL101(Fe) and Bi_2_O_3_ [57], which is consistent with the results from PL spectra and photocurrent analysis. In summary, PL spectra, photocurrent responses, and EIS analysis all indicated that Bi_2_O_3_/MIL101(Fe) heterojunctions can result in more effective electron–hole pair separation, higher interfacial electron transfer rate, and thus weakened charge transfer resistance, which may facilitate the improvement of Bi_2_O_3_/MIL101(Fe) photocatalytic activity.

### Photocatalytic degradation of chlortetracycline

**Figure 6:**
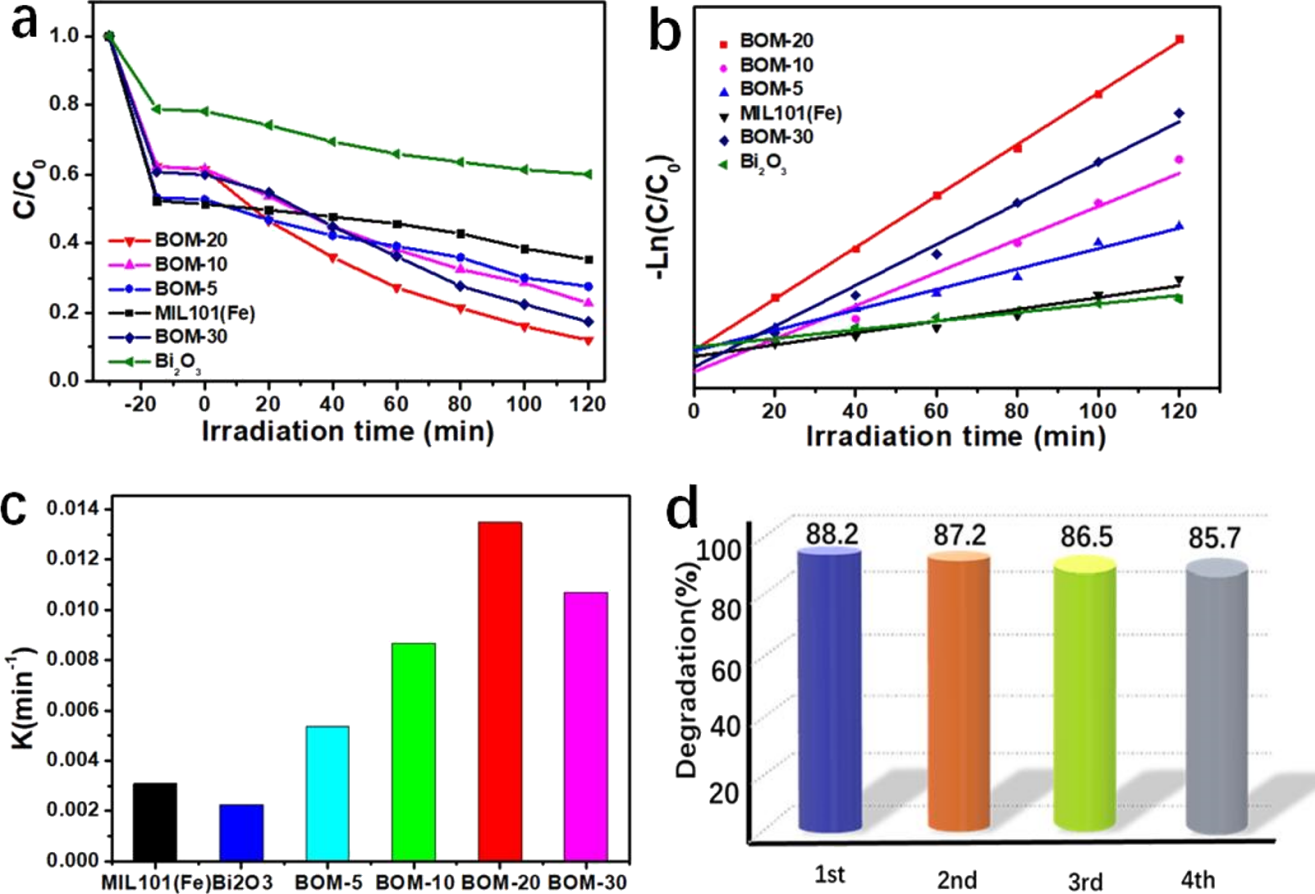
(a) Photodegradation of CTC over as-prepared photocatalysts under visible irradiation (λ > 420 nm). (b) Quasi-first-order kinetic curve. (c) Apparent rate constants (*k*) and (d) degradation rate of CTC in a four-cycle test of the BOM-20 photocatalyst.

To evaluate the photocatalytic activity of BOM-20, the photodegradation of chlortetracycline (CTC) under visible-light irradiation was conducted. It can be seen from Figure 6a that Bi_2_O_3_ shows poor adsorption and photocatalytic activity, and the CTC removal rate is only 40% within 120 min. MIL101(Fe) shows strong adsorption capacity in the dark and a poorer photocatalytic activity. With the photocatalysis of MIL101(Fe), the photodegradation efficiency of CTC only could reach 64.5% after irradiation for 120 min. Compared with MIL101(Fe), Bi_2_O_3_/MIL101(Fe) composites exhibit a slightly weakened adsorption efficiency for CTC, which is perhaps due to decreased specific surface areas relative to MIL101(Fe) (Supporting Information File 1, Figure S1, Table S1). However, the photocatalytic activity of the Bi_2_O_3_/MIL101(Fe) composite is substantially higher than that of pristine Bi_2_O_3_ and MIL101(Fe). Among various composites, BOM-20 shows the highest activity with a CTC removal rate up to 88.2%. Additionally, Figure 6b demonstrates that the photocatalytic degradation of CTC accords with the quasi-first-order kinetic model. Figure 6c shows the apparent kinetic constant (*k*) of CTC photodegradation by the as-prepared catalysts. The *k* value of BOM-20 (0.01348 min^−1^) is the largest, which is 5.9 and 4.3 times higher than that of Bi_2_O_3_ (0.00226 min^−1^) and MIL101(Fe) (0.00309 min^−1^), respectively. The enhanced photocatalytic activity is attributed to the effective separation and migration of the photoinduced charge carriers due to the formation of the Bi_2_O_3_/MIL101(Fe) heterojunction and the enhanced visible-light absorption. In addition, some recently reported research results of photocatalytic CTC degradation are shown in Supporting Information File 1, Table S2. The CTC degradation efficiency of the as-prepared BOM-20 composite in this work is significantly satisfactory.

The stability of BOM-20 for the degradation of CTC was examined via cycling degradation tests. As depicted in Figure 6d, BOM-20 exhibits no obvious loss of activity after four consecutive cycles. A slight decrease in CTC degradation rate is perhaps caused by an inevitable loss of the photocatalyst during recycling. As a result, the BOM-20 heterojunction possesses excellent stability in the photocatalytic reaction. Moreover, in the XRD patterns of fresh BOM-20 and BOM-20 after the photocatalytic cyclic test (Supporting Information File 1, Figure S2), no obvious change was found, which suggests that the crystal structure of BOM-20 remained unchanged after the photocatalytic reaction. This result further confirms the stability of the BOM-20 heterojunction as a photocatalyst.

**Figure 7:**
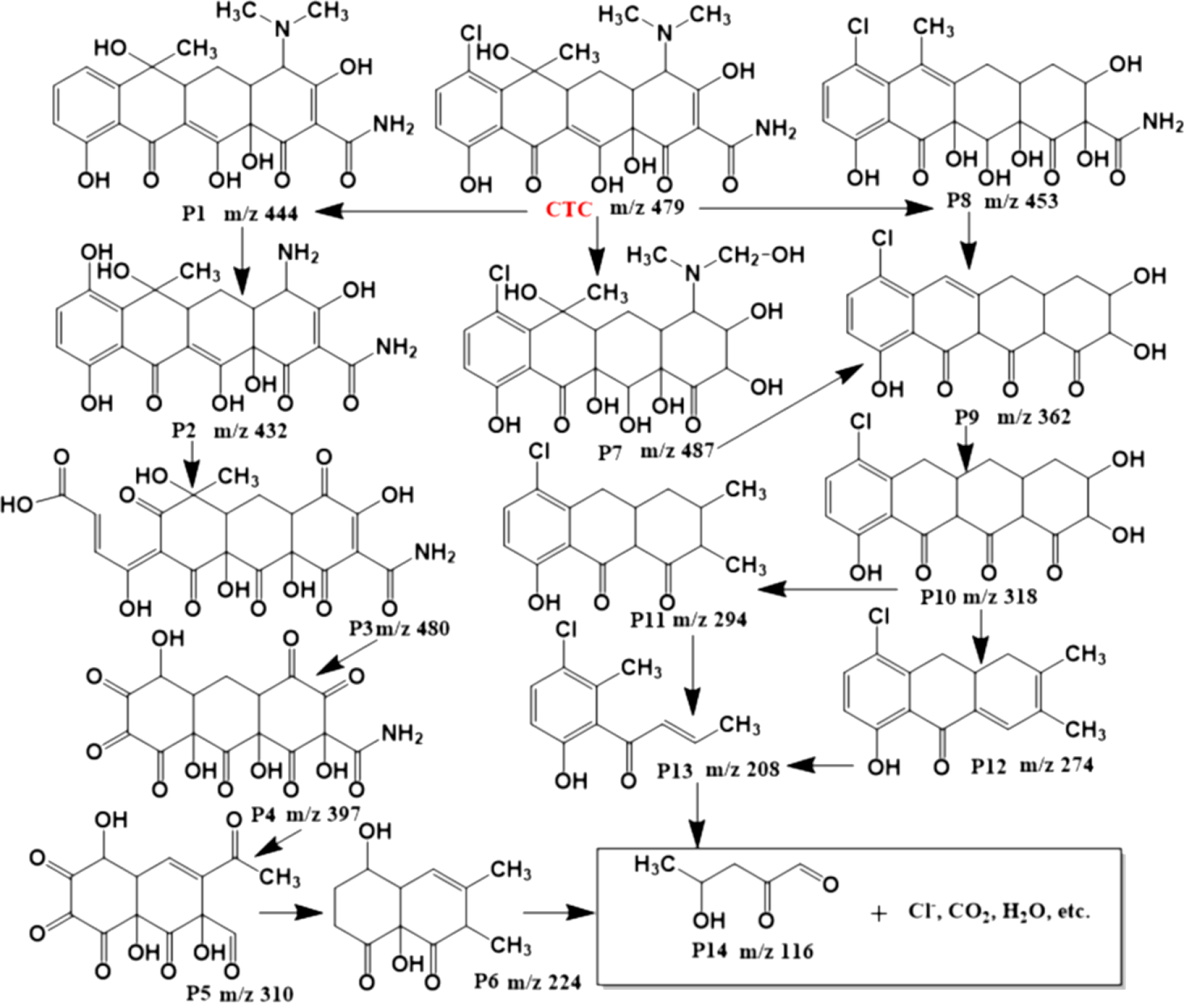
Possible degradation pathways of CTC catalyzed by BOM-20.

To explore the CTC degradation pathway with the existence of a BOM-20 heterojunction, the intermediate products in CTC degradation were detected by liquid chromatography–mass spectrometry (LC–MS), and the mass spectra are showed in Supporting Information File 1, Figure S3. Based on analysis of intermediates, the possible degradation pathways were speculated and shown in Figure 7. Two possible degradation pathways were deduced through hydroxylation, dehydration, and ring opening [59]. First, dechlorination of CTC due to the attack of radicals resulted in the formation of P1 intermediates with *m*/*z* of 444. Then P2 (*m*/*z* 432) was obtained from P1 through the loss of an *N*-methyl group and hydroxylation [4]. The P2 was in turn decomposed into possible intermediates (P3, P4, P5, P6) via ring opening, ketonization/dehydroxylation, deamidation, and dialdehyde [11,60]. The second pathway was initiated from deamidation or deamination of CTC. The P7 (*m*/*z* 486) was obtained from deamidation and hydroxylation of CTC. Meanwhile, CTC was decomposed into P8 (*m*/*z* 453) through deamination and hydroxylation. Then, the produced P7 and P8 were converted to P9 (*m*/*z* 362) through deamidation, ketonization/dihydroxylation, and demethylation [3]. The P10 (*m*/*z* 318) was derived from P9 due to the loss of hydroxyl and ketone groups and was further decomposed into P11 (*m*/*z* 294) or P12 (*m*/*z* 274). The P11 and P12 were further decomposed into P13 (*m*/*z* 208) by double-bond break and ring-opening reaction [1]. Subsequently, with the attack of active species, intermediates (P6, P13) are further oxidized and eventually evolve into possible small molecular intermediates such as (P14 *m*/*z* 116), Cl^−^, CO_2_, and H_2_O [60].

### Proposed charge-transfer mechanism

To explore charge-transfer mechanisms, the main active species involved in degradation of CTC catalyzed by BOM-20 was investigated by radical trapping test. Ethylenediaminetetraacetic acid disodium (EDTA-2Na), isopropanol (IPA), and vitamin C (VC) were used as scavengers for holes (h^+^), hydroxyl radicals (•OH), and superoxide radicals (•O_2_^−^), respectively.

**Figure 8:**
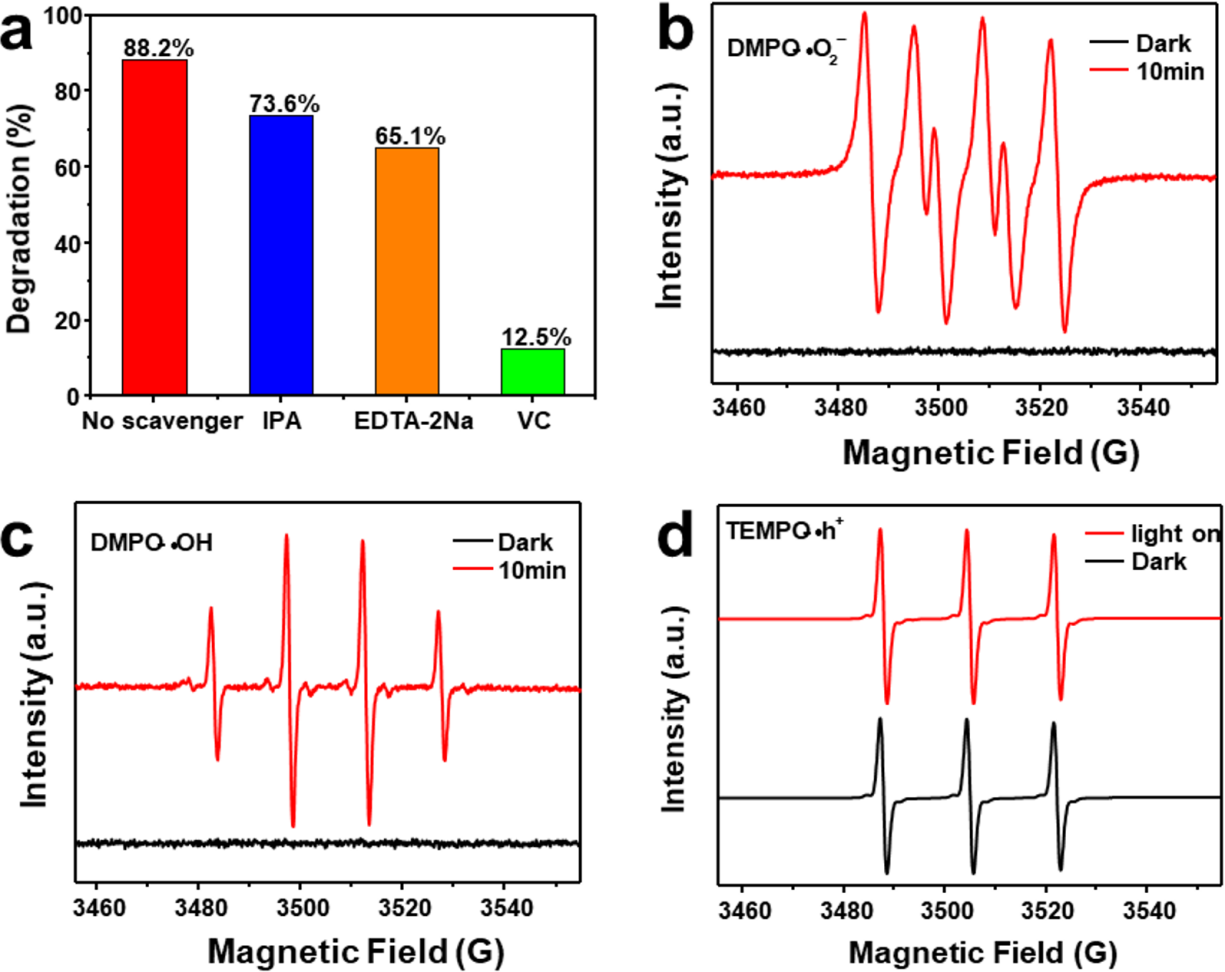
(a) The degradation efficiency of CTC in the presence of different scavengers. ESR spectra of (b) DMPO •O_2_^−^, (c) DMPO •OH, and (d) TEMPO •h^+^ for the BOM-20 photocatalyst.

Figure 8a shows the degradation rate of CTC in the presence of three scavengers. As shown in Figure 8a, when VC was added to the photocatalytic reaction system, the degradation efficiency of CTC significantly decreased from 88.2% to 12.5%, which revealed that •O_2_^−^ is main active species in the degradation of CTC. In addition, after the addition of IPA and EDTA-2Na, the degradation efficiency of CTC decreased to 73.6% and 65.1%, respectively, indicating that h^+^ and •OH also played a role in the photocatalytic process to some extent. To further confirm the free radicals produced in the photocatalytic degradation of CTC, ESR spectra of the samples were measured. As given in Figure 8b,c, no signals of •O_2_^−^ and •OH were found in dark conditions. However, the strong characteristic signals of •O_2_^−^ and •OH were detected after visible-light irradiation for 10 min, which demonstrated that •O_2_^−^ and •OH were produced during the photodegradation process. Figure 8d shows that the signal of h^+^ in the dark is stronger than that under visible-light irradiation, which indicates that h^+^ plays a role in photocatalytic reaction progress. This result is in accordance with that of the radical trapping test.

**Figure 9:**
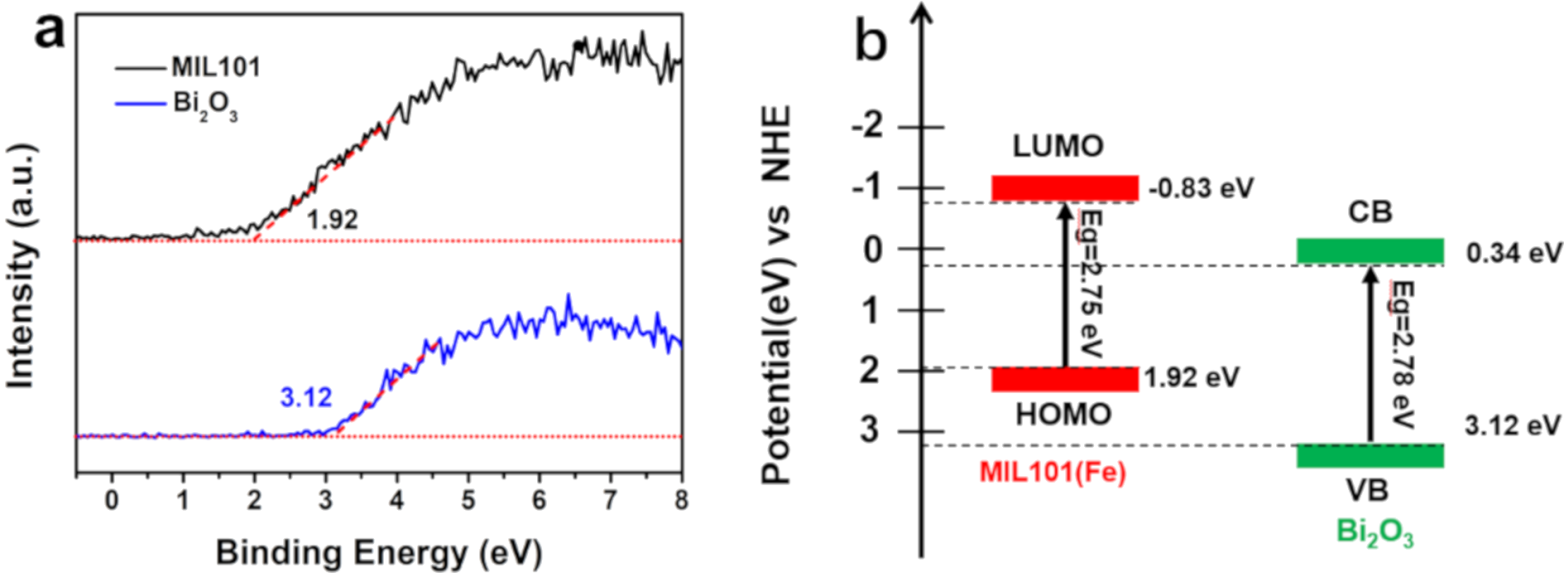
(a) The VB-XPS spectra and (b) the energy band position of Bi_2_O_3_ and MIL101(Fe).

**Figure 10:**
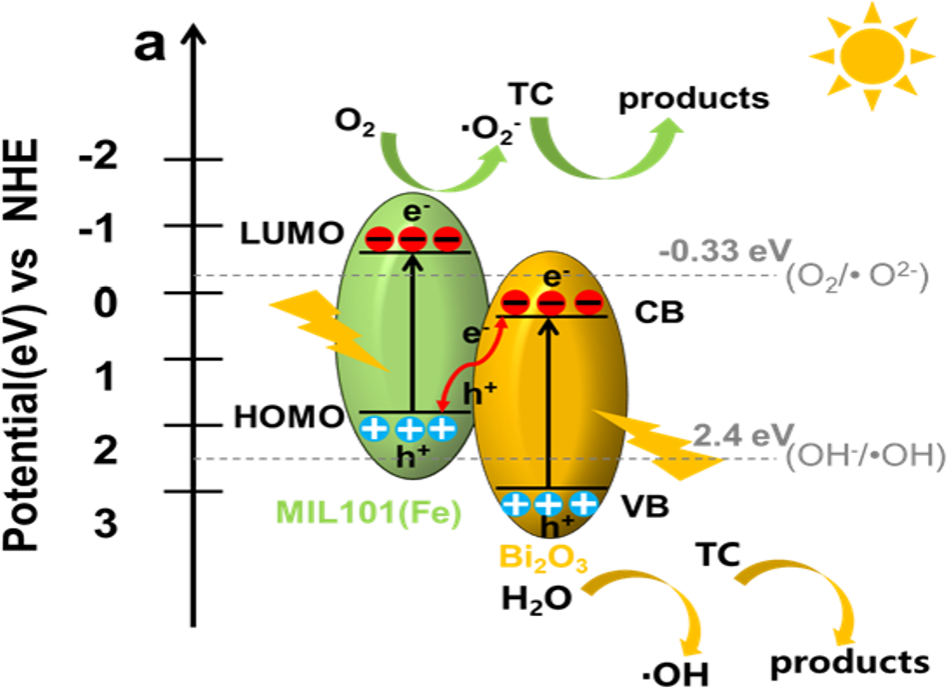
Z-Scheme charge-transfer mechanism of a BOM-20 heterojunction.

In order to account for the formation of the active species and reveal the photocatalytic mechanism, the band structure was measured by UV–vis DRS and valence band X-ray photoelectron spectroscopy (VB-XPS). UV–vis DRS spectra and the bandgap (*E*_g_) of Bi_2_O_3_ and MIL101(Fe) were discussed in Figure 5a, and the obtained bandgap of Bi_2_O_3_ and MIL101(Fe) are 2.78 and 2.75 eV, respectively. Figure 9a shows the VB-XPS spectra of Bi_2_O_3_ and MIL101(Fe). As seen in Figure 9a, the valence band potential (*E*_VB_) of Bi_2_O_3_ and the highest occupied molecular orbital (HOMO) potential of MIL101(Fe) are 3.12 and 1.92 eV, respectively. According to the equation: *E*_g_ = *E*_VB_ − *E*_CB_, the conduction band potential (*E*_CB_) of Bi_2_O_3_ and the lowest unoccupied molecular orbital (LUMO) potential of MIL101(Fe) are calculated as 0.34 and −0.83 eV, respectively. The energy band position of Bi_2_O_3_ and MIL101(Fe) are shown in Figure 9b. According to Figure 9b, *E*_CB_ and *E*_VB_ of Bi_2_O_3_ are more positive than the LUMO and HOMO potentials of MIL101(Fe), respectively, which suggests that possible charge-transfer mechanisms on the interface of Bi_2_O_3_/MIL101(Fe) heterojunction may be conventional type-II charge transfer or Z-scheme charge transfer. For the type-II charge-transfer mechanism, photogenerated electrons (e^−^) tend to transfer from the LUMO of MIL101(Fe) to the conduction band (CB) of Bi_2_O_3_, while h^+^ in the valence band (VB) of Bi_2_O_3_ will migrate to the HOMO of MIL101(Fe). Resultantly, photogenerated e^−^ mainly accumulate in the CB of Bi_2_O_3_. Nevertheless, the *E*_CB_ (0.34 eV) of Bi_2_O_3_ is more positive than the potential of O_2_/•O_2_^−^ (−0.33 eV), so •O_2_^−^ cannot be produced. Furthermore, the HOMO potential (1.92 eV) of MIL101(Fe) is more negative than the potential of H_2_O/•OH (2.4 eV), indicating that the generation of •OH is unlikely. Obviously, the hypothesis of type-II charge transfer is contradictory to the results from radical trapping and ESR experiments. Thus, the Z-scheme charge-transfer model is expected to clarify the photocatalytic mechanism of BOM-20 for degradation of CTC. As illustrated in Figure 10, under visible-light irradiation, MIL101(Fe) and Bi_2_O_3_ can be excited to produce e^−^ and corresponding h^+^, respectively. Due to the internal field at the interface of the heterojunction, the photogenerated electrons in the CB of Bi_2_O_3_ transfer to the VB of MIL101(Fe) and quickly recombine with the holes of MIL101(Fe), which boosts interfacial separation of charge carriers. As a result, e^−^ and h^+^ are reserved in the CB of MIL101(Fe) and in the VB of Bi_2_O_3_, respectively. Since the *E*_CB_ (−0.83 eV) of MIL101(Fe) is more negative than the reduction potential of O_2_/•O_2_^−^ (−0.33 eV), the e^−^ in LUMO of MIL101(Fe) can capture O_2_ in solution to generate •O_2_^−^. Similarly, the h^+^ in the VB of Bi_2_O_3_ may oxidize H_2_O to produce •OH due to the fact that the VB potential (3.12 eV) of Bi_2_O_3_ is more positive than the redox potential of H_2_O/•OH (2.4 eV). The produced •O_2_^−^ and •OH decompose CTC. Additionally, the h^+^ in the VB of Bi_2_O_3_ can directly participate in CTC degradation. These results are in good agreement with the analysis of the trapping test and ESR spectra. Therefore, the Z-scheme charge-transfer pathway is confirmed in the Bi_2_O_3_/MIL101(Fe) heterojunction. Apparently, the Z-scheme heterojunction is conducive to the effective separation of photogenerated carriers. Furthermore, h^+^ with higher oxidizing ability and e^−^ with stronger reducing ability participate in the redox reaction and production of •OH and •O_2_^−^. Accordingly, the significant improvement of the photocatalytic activity of Bi_2_O_3_/MIL101(Fe) can be attributed to the formation of a Z-scheme heterojunction.

## Conclusion

In summary, a novel heterojunction photocatalyst composed of 0D Bi_2_O_3_ nanoparticles and 3D spindle-shaped MIL101(Fe) was prepared via a facile solvothermal method. Compared with pure Bi_2_O_3_ and MIL101(Fe), the BOM-20 composite with optimal Bi_2_O_3_-to-MIL101(Fe) ratio shows significantly enhanced photocatalytic activity for degradation of CTC under visible-light irradiation, the removal of CTC reaches nearly 88.2% within 120 min. The excellent photocatalytic performance of the BOM-20 composite was attributed to a Z-scheme heterojunction between Bi_2_O_3_ and MIL101(Fe). The construction of the Z-scheme heterojunction not only broadened the visible light absorption range of the Bi_2_O_3_/MIL101(Fe) composite but also realized the effective transfer of photogenerated charge carriers and retained a high redox capacity of photogenerated electrons and holes. Additionally, through the identification of intermediate products by LC–MS, the photocatalytic degradation pathway of CTC was speculated. This work provides an idea for designing and synthesizing MOF-based Z-scheme heterojunction photocatalysts.

## Supporting Information

File 1 Additional figures and tables.
