# Supplementary material for: Spindle-like MIL101(Fe) decorated with Bi2O3 nanoparticles for enhanced degradation of chlortetracycline under visible-light irradiation
Source: Beilstein J Nanotechnol. 2022 Sep 28;13:1038–50. doi: 10.3762/bjnano.13.91 (PMC9531557; doi:10.3762/bjnano.13.91)
Supplement: File 1 — Additional figures and tables. [file Beilstein_J_Nanotechnol-13-1038-s001.pdf]

BEILSTEIN JOURNAL OF NANOTECHNOLOGY

Supporting Information

for

Spindle-like MIL101(Fe) decorated with Bi_2O_3 nanoparticles for enhanced degradation of chlortetracycline under visible-light irradiation

Chen-chen Hao, Fang-yan Chen, Kun Bian, Yu-bin Tang and Wei-long Shi

Beilstein J. Nanotechnol. **2022,** *13,* 1038–1050. doi:10.3762/bjnano.13.91

Additional figures and tables

License and Terms: This is a supporting information file under the terms of the Creative Commons Attribution License (https://creativecommons.org/licenses/by/4.0). Please note that the reuse, redistribution and reproduction in particular requires that the author(s) and source are credited and that individual graphics may be subject to special legal provisions.
The license is subject to the *Beilstein Journal of Nanotechnology* terms and conditions: (https://www.beilstein-journals.org/bjnano/terms)

Table S1: BET surface area, pore volume, and pore size of Bi_2O_3, MIL101(Fe), and and BOM-20.

Samples	S_{BET} (m^2/g)	V_p (cm^3/g)	Pore size (Å)
MIL101(Fe)	276.35	0.42	25.64
Bi_2O_3	6.19	0.01	62.6
BOM-20	60.23	0.12	47.8

Table S2: Comparison of photocatalytic efficiencies of other previously reported photocatalysts for degradation of CTC in recent years.

**Photocatalysts**	**Dosage of catalyst (g/L)**	**CTC concentration (mg/L)**	**Reaction time (min)**	**Degradation rate (%)**	**Ref.**
**Ce-MOF/GO/Fe_3O_4**	0.45	20	180	80.5	[S1]
**$Zn_{0.75}Mn_{0.75}Fe_{1.5}O_4$/$ZnFe_2O_4$/ZnO**	0.4	10	120	61.9	[S2]
**CNPs**	0.1	10	120	82.2	[S3]
**3Co/0.3$ZnIn_2S_4$/GaN**	0.4	10	120	81.0	[S4]
**$Bi_{1.81}MnNbO_{6.72}$/sulfite**	0.3	20	120	76.2	[S5]
**Co_2SnO_4-SnO_2/GC**	0.4	10	80	83.0	[S6]
**BP-$BiVO_4$**	0.15	10	120	88.0	[S7]
**BOM-20**	**0.3**	**20**	**120**	**88.2%**	**this work**

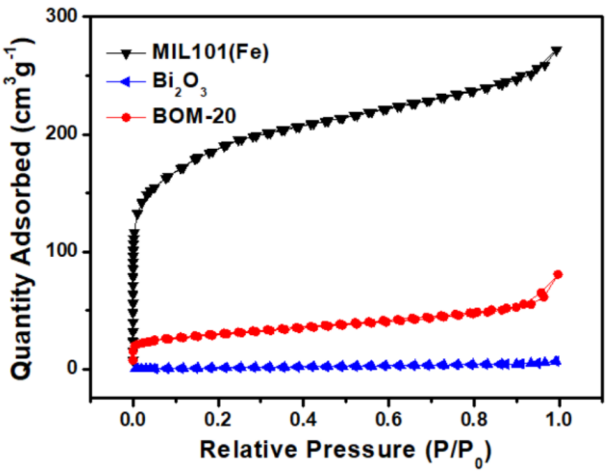

Figure S1: N_2 adsorption–desorption isotherms.

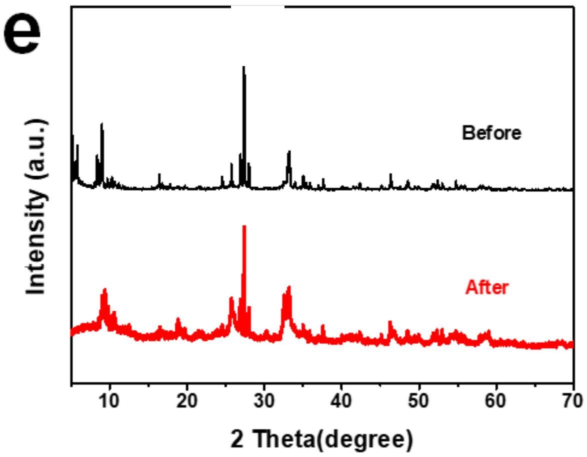

Figure S2: XRD patterns of the BOM-20 before and after the cyclic test.

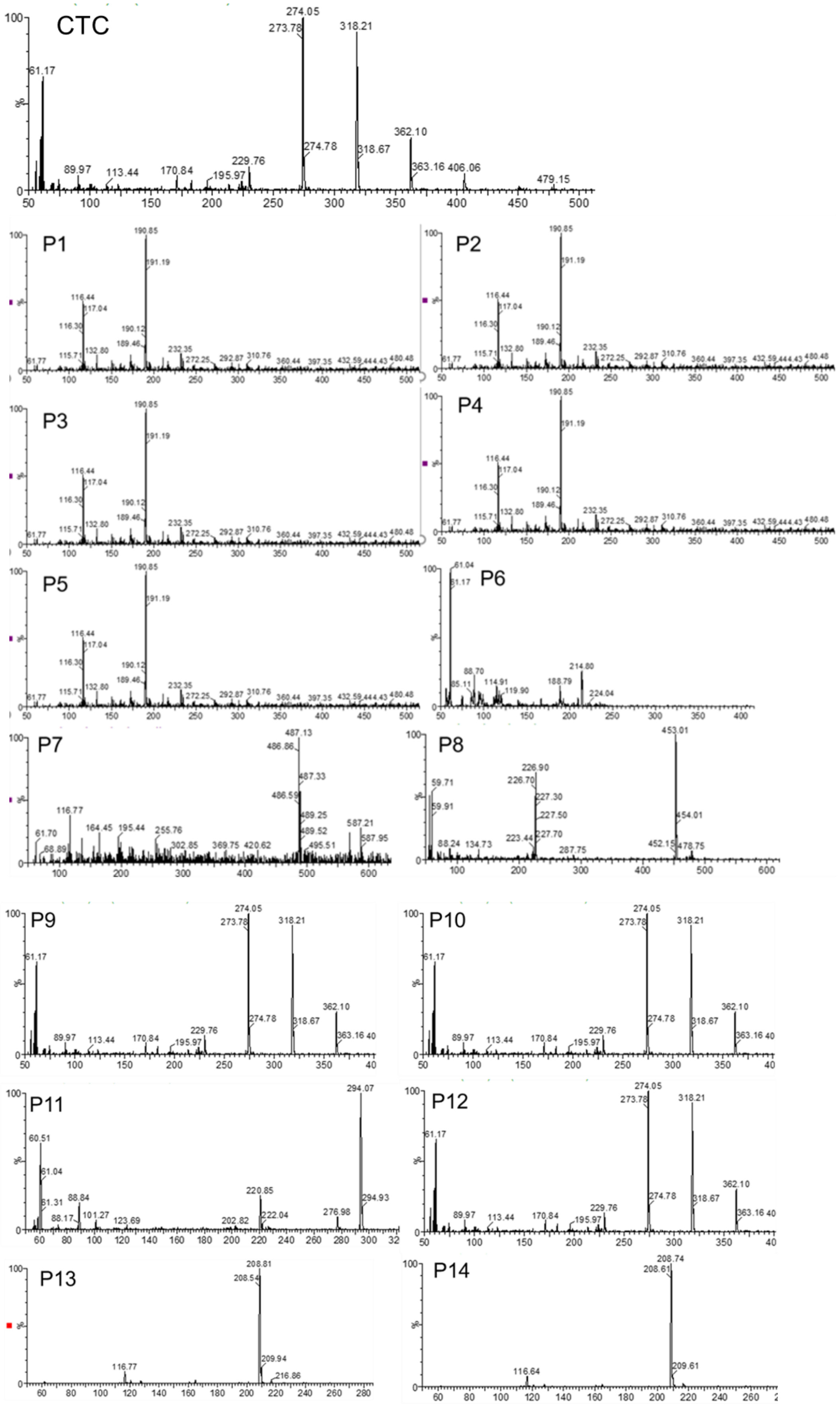

Figure S3: Liquid chromatography–mass spectroscopy results of TCT and intermediates.

References

[S1] Y. Bai, M. Han, X. Li, S. Feng, L. Lu, S. Ma, Facile and efficient photocatalyst for degradation of chlortetracycline promoted by H_2O_2. Inorg. Chem. Front. **2022**, 9, 2952-2963.

[S2] Fu, J., Yu, X., Li, Z. et al. Study on the degradation of chlortetracycline hydrochloride in mariculture wastewater by $Zn_{0.75}Mn_{0.75}Fe_{1.5}O_4/ZnFe_2O_4/ZnO$ photocatalyst. Water Air Soil Pollut. **2021**, 232, 12.

[S3] X. Liu, W. Hou, Y. Huang, H. Zhao, Z. Song, Y. Huang, Facile and green synthesis of carbon nanopinnacles for the removal of chlortetracycline: Performance, mechanism and biotoxicity. Chem. Eng. J., **2022**, 433, 133822.

[S4] Y. He, J. Shi, Q. Yang, Y. Tong, Z. Ma, L. B. Junior, B. Yao, Co-doped 3D petal-like $ZnIn_2S_4/GaN$ heterostructures for efficient removal of chlortetracycline residue from real pharmaceutical wastewater. Chem. Eng. J., **2022**, 446, 137355.

[S5] J. Zhao, Q. Song, Q. He, D. D. Dionysiou, F. Wu, Y. Feng, X. Zhang. Fabrication of $Bi_{1.81}MnNbO_{6.72}$/sulfite system for efficient degradation of chlortetracycline. Chemosphere. **2021**, 268, 129269.

[S6] J. Zheng, L. Zhang, Rational design and fabrication of multifunctional catalyzer Co_2SnO_4-SnO_2/GC for catalysis applications: photocatalytic degradation/catalytic reduction of organic pollutants. Appl. Catal. B: Environ., **2018**, 231, 34-42.

[S7] M. Zhang, Q. Shi, X. Cheng, et al., Accelerated generation of hydroxyl radical through surface polarization on $BiVO_4$ microtubes for efficient chlortetracycline degradation. Chem. Eng. J., **2020**, 400, 125871.
